# Optically Responsive Protein Coating of DNA Origami for Triggered Antigen Targeting

**DOI:** 10.1021/acsami.2c10058

**Published:** 2022-08-19

**Authors:** Iris Seitz, Heini Ijäs, Veikko Linko, Mauri A. Kostiainen

**Affiliations:** † Department of Bioproducts and Biosystems, Aalto University, P.O. Box 16100, 00076 Aalto, Finland; ‡ Ludwig-Maximilians-University, Geschwister-Scholl-Platz 1, 80539 Munich, Germany; ¶ LIBER Center of Excellence, Aalto University, P.O. Box 16100, 00076 Aalto, Finland

**Keywords:** DNA nanotechnology, protein coating, photoresponsiveness, antigen targeting, electrostatic binding

## Abstract

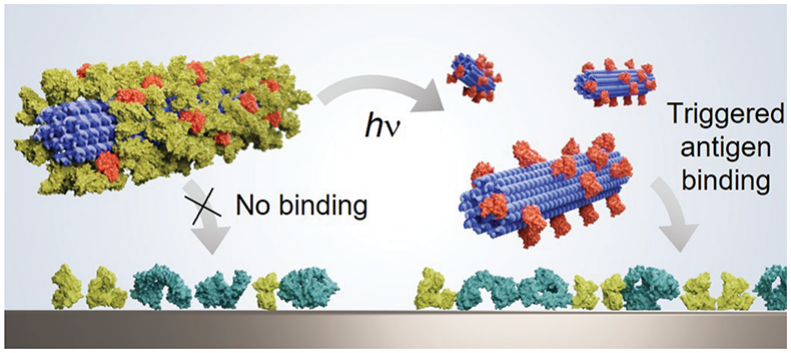

DNA nanostructures have emerged as modular building blocks in several research fields including biomedicine and nanofabrication. Their proneness to degradation in various environments has led to the development of a variety of nature-inspired protection strategies. Coating of DNA origami nanostructures with proteins can circumvent degradation and alter their properties. Here, we have used a single-chain variable antibody fragment and serum albumin to construct positively charged and stimuli-responsive protein-dendron conjugates, which were complexed with DNA origami through electrostatic interactions. Using a stepwise assembly approach, the coated nanostructures were studied for their interaction with the corresponding antigen in fluorescence-based immunoassays. The results suggest that the antibody–antigen interaction can be disturbed by the addition of the bulky serum albumin. However, this effect is fully reversible upon irradiation of the structures with an optical stimulus. This leads to a selective dissociation of the serum albumin from the nanostructure due to cleavage of a photolabile group integrated in the dendron structure, exposing the antibody fragment and enabling triggered binding to the antigen, demonstrating that serum albumin can be considered as an externally controlled “camouflaging” agent. The presented stimuli-responsive complexation approach is highly versatile regarding the choice of protein components and could, therefore, find use in DNA origami protection, targeting, and delivery as well as their spatiotemporal control.

## Introduction

Over the past decades, structural DNA nanotechnology has developed into a noteworthy research field.^1−3^ With the invention of the DNA origami technique,^4,5^ facile production of DNA nanostructures and, therefore, also the realm of custom DNA nanodesigns have become widely accessible.^6^ With the help of short single-stranded “staple” strands, a long, single-stranded “scaffold” strand is thereby self-assembled into higher order structures.^4^ Although derived from only a few different scaffold sequences, a great variety of DNA origami structures has been presented, ranging from elementary 2D and 3D shapes^4,7−10^ to more complex structures with twists and curves^11,12^ and meshed constructions with automated design.^13−15^

The user-defined design and the high addressability allow for the utilization of these structures in a wide range of applications, including nanoelectronics,^16^ nanorobotics,^17,18^ bottom-up nanofabrication,^19^ as well as biosensing and biomedicine.^20,21^ For biomedical applications, several DNA nanostructures have been further functionalized with antibodies, affibodies, and aptamers for targeting purposes.^22^ These targeting agents can be site-specifically attached, ensuring an optimal interaction with their receptor which was found to be dependent on the origami shape and orientation.^23^ Antibody–antigen interactions can be furthermore studied by immobilization of small-molecule antigens onto the DNA origami surface^24^ and can be applied for triggering a conformation change of the origami which is exploited for cargo display, such as drug molecules.^25,26^ Currently, targeting is widely employed for the development of DNA-based tools for treatment of cancer. Several studies show enhanced inhibition of malignant cell growth, demonstrating efficient drug delivery.^22^

Regardless of the area of application, the intactness of the structures is of utmost importance. However, their structural integrity can be compromised in demanding environments. These include low-cation buffers,^27^ high temperatures,^28^ and physiological conditions^29,30^ including nuclease-rich media.^29,31,32^ The overall stability has been found to be dependent on the design and shape of the DNA superstructure.^27,29,33−35^

The aim to increase the stability of DNA nanostructures has yielded a variety of coating strategies: The high addressability of the surface of the nanostructures allows for the precise arrangement of nucleic acid-functionalized biomolecules, such as lipids^36−38^ and proteins,^24,39−41^ resulting in highly ordered supramolecular assemblies.

Because of the high net negative charge of the DNA origami originating from the phosphate groups in the backbone, these structures are apt to serve as templates for positively charged building blocks through electrostatic interactions. These include intrinsically charged compounds, such as virus capsid proteins that may enhance the delivery of DNA origami through encapsulation,^42^ and cationic lipid coatings that are shown to increase stability against DNase I digestion.^43^ In addition, there are a plethora of attractive options based on cationic polymers that can be harnessed in attaching favored molecules to DNA nanostructures.^44−47^ Furthermore, polyethylene glycol (PEG) oligolysine coating was found not only to increase the stability of DNA origami in low-cation buffers^46^ but also to protect the structures against enzymatic degradation.^46,47^

Another example of electrostatic coating strategies was presented by Auvinen et al., who used a Newkome-type dendron containing spermine groups as the positive counterpart which was conjugated to bovine serum albumin (BSA). By mixing the protein-dendron conjugate with brick-shaped DNA origami, a uniform protein coating was obtained. The coated origami elicited enhanced stability against DNase I digestion and improved cell transfection efficiency. Importantly, a notably decreased immune response was reported after BSA coating, thus underlining the versatility and immunocompatibility achieved through this protection scheme.^48^

Even though these components advance the properties of the nanostructures, the resulting coatings are mainly static. Establishing systems responsive to external stimuli would be advantageous, especially with regards to therapeutic applications. Responsiveness could allow multiple/continuous treatment due to the addition and removal of the stimuli and, more importantly, a strict control of the stimuli’s intensity and location, which particularly applies for optical irradiation.^49^ Light is a versatile stimuli that has previously been used for nanoparticles to induce both controlled drug release and precise targeting by photocleavage of, for instance, shielding ligands.^50^

**Figure 1:**
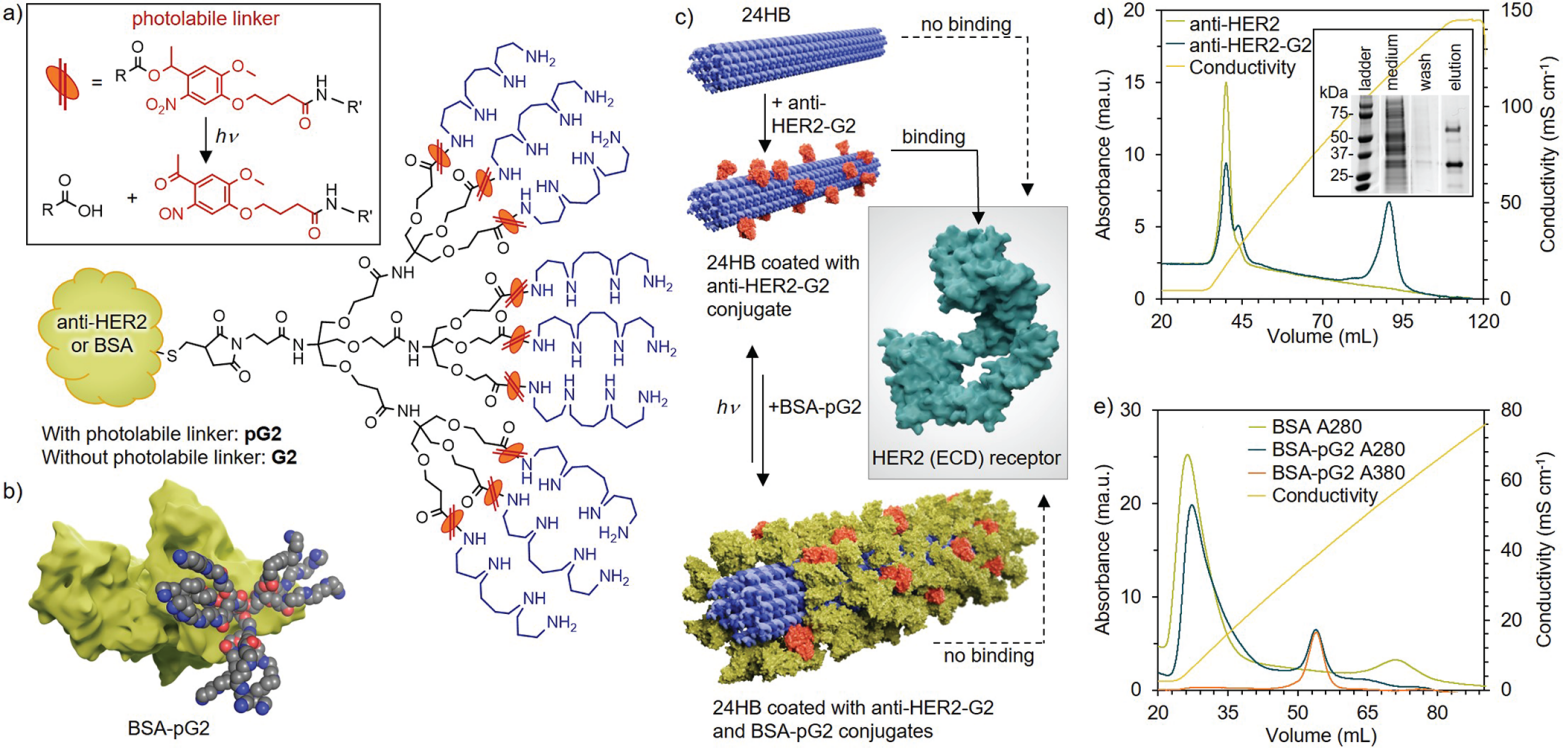
Schematics of the photolabile dendron coating of DNA origami. (a) Bovine serum albumin (BSA) and anti-human epidermal growth factor 2 single-chain antibody fragment (anti-HER2) are conjugated to a second generation Newkome-type dendron (G2) via cysteine-maleimide coupling. For BSA, the dendron contains a photolabile *o*-nitrobenzyl group (inset; BSA-pG2). (b) Model of the structure of BSA-pG2. (c) Electrostatic interactions facilitate the protein-dendron conjugate assembly on a rod-shaped 24 helix-bundle (24HB) DNA origami. A two-component coating is applied sequentially, and the binding properties to the antigen (extracellular domain of HER2) are investigated before and after irradiation with ultraviolet (UV) light. (d) Preparation and purification of anti-HER2-G2 which was expressed recombinantly in *E. coli* (polyacrylamide gel electrophoresis (PAGE), inset) and purified from unbound anti-HER2 after conjugation by cation exchange fast protein liquid chromatography (FPLC). (e) Purification of BSA-pG2 from unbound BSA after conjugation by cation exchange FPLC.

Here, we present a two-component protein coating strategy for equipping DNA origami surfaces with both targeting and camouflaging proteins. To achieve antigen targeting, we have coupled a single-chain variable antibody fragment to highly positively charged dendron structures which electrostatically bind to DNA origami. In a BSA-dendron complex, each dendron branch in a positively charged DNA-binding domain contains a photolabile group that can be cleaved upon mild ultraviolet (UV) light exposure (see Figure 1).

For investigating the impact of the individual compounds as coating and targeting agents on a rod-shaped DNA origami model structure, we have established a fluorescence-based plate immunoassay. By optimizing the ratio of the protein-based compounds used compared to the DNA origami, we showed that the bulky BSA can act as a “camouflaging” agent by prohibiting the binding of the antibody coated DNA origami to its corresponding antigen. Upon exposure to UV light, BSA is released from the structure and binding is enabled.

## Results and Discussion

We have used two types of multivalent, second generation Newkome-type dendrimers (see Figure 1a). Both dendrons contain positively charged (+27) spermine groups (shown in blue) and a core *N*-maleimido group; however, they only differ in the linker between the frame and the spermine surface groups (see Figure 1a, inset). The dendron types with or without the photolabile *o*-nitrobenzyl group (shown in orange) are termed pG2 and G2, respectively. The *o*-nitrobenzyl group is responsive to light (at λ = 365 nm), and the irradiation will result in cleavage of the group, thus separating the positively charged DNA binding domain and the core structure.^51^ Both dendron types have been described to have excellent DNA binding properties and allow facile adhesion of proteins solely based on electrostatic interactions.^48,51,52^

For the coupling of proteins and dendrons, an *N*-maleimido group and a free cysteine sulfhydryl group were reacted at ambient conditions to form a covalent bond.^52^ The BSA (molecular weight ∼66.4 kDa), acting as the main bulky coating component, has a single solvent-exposed cysteine residue (Cys34) and is, therefore, readily accessible for conjugation. For proof-of-principle targeting purposes, we selected the human epidermal growth factor receptor 2 (HER2). The HER2 receptor is a membrane tyrosine kinase important for promotion of cell proliferation and was found to be overexpressed in ∼20% of breast cancers, which makes it suitable for targeted treatment.^53^

However, instead of using monoclonal antibodies as its counterpart, engineered antibody fragments, such as single-chain antibody fragments, may usually be the preferred choice in several applications.^54^ Especially, their smaller size is particularly advantageous in our two-component protein coating setting. To make it suitable for site-specific conjugation to the dendron, this highly engineered protein fragment contains an artificial C-terminal cysteine residue (scFv^C^, Cys257). For simplicity, the antibody fragment will be named as anti-HER2.

While the anti-HER2 was conjugated to the non-photoresponsive dendron type (anti-HER2-G2), BSA was predominantly used with the photolabile dendron (BSA-pG2, for the schematic see Figure 1b). Serving as a negative, nonresponsive control, BSA was also conjugated to the non-photodegradable dendron (BSA-G2).

The highly positive charge of the dendrimers allows electrostatic interaction with the negatively charged DNA origami (see Figure 1c). As a model, the rod-shaped 24-helix bundle DNA origami (24HB dimensions: diameter 16 nm, length 107 nm; see Figure S1) was used.^55^ The structure was prepared from the p7560 scaffold, and it was based on a honeycomb lattice geometry. Additionally, ATTO488-functionalized strands (A488, 24 fluorescence dye molecules per origami) were integrated into the structure by hybridization to staples containing a 3′-overhang. For coating purposes, the DNA origami structure was first complexed with the antibody conjugate followed by an incubation with the BSA conjugate. The binding properties of the nanostructure at different coating stages to the extracellular domain (ECD) of HER2 were investigated in a fluorescence-based plate immunoassay.

The anti-HER2 (molecular weight 27.6 kDa) was expressed recombinantly in *Escherichia coli* RV308 cells and purified from the growth media using His-beads. The proteins present in different steps of the expression and purification were monitored by polyacrylamide gel electrophoresis (PAGE, see Figure 1d, inset). The conjugation reaction of anti-HER2 and G2 was performed with an excess of G2, while for obtaining BSA-G2 and BSA-pG2 the dendron was chosen to be the limiting factor. Subsequently, the protein-dendron conjugate was separated from excess compounds by fast protein liquid chromatography (FPLC) using a heparin column. The conjugates were eluted from the column by applying a NaCl gradient. Both unconjugated anti-HER2 and BSA show a small affinity toward heparin (Figure 1d at roughly 40 mL for anti-HER2, green, and Figure 1e and Figure S2 at roughly 25–30 mL for BSA, green). However, an additional peak (90 mL, Figure 1d, and 55 mL, Figure 1e) is observed, indicating a successful conjugation of the proteins with the corresponding dendron. The presence of the photolabile dendron on BSA-pG2 could be furthermore monitored from the absorbance signal at 380 nm originating from the *o*-nitrobenzyl group. It can be noted that the dendron type has an influence on the affinity toward heparin. While a conductivity of ∼100 mS cm^–1^ was necessary to elute G2 conjugates, BSA-pG2 required only 55 mS cm^–1^.

### Interaction between Free Antibody and Antigen

**Figure 2:**
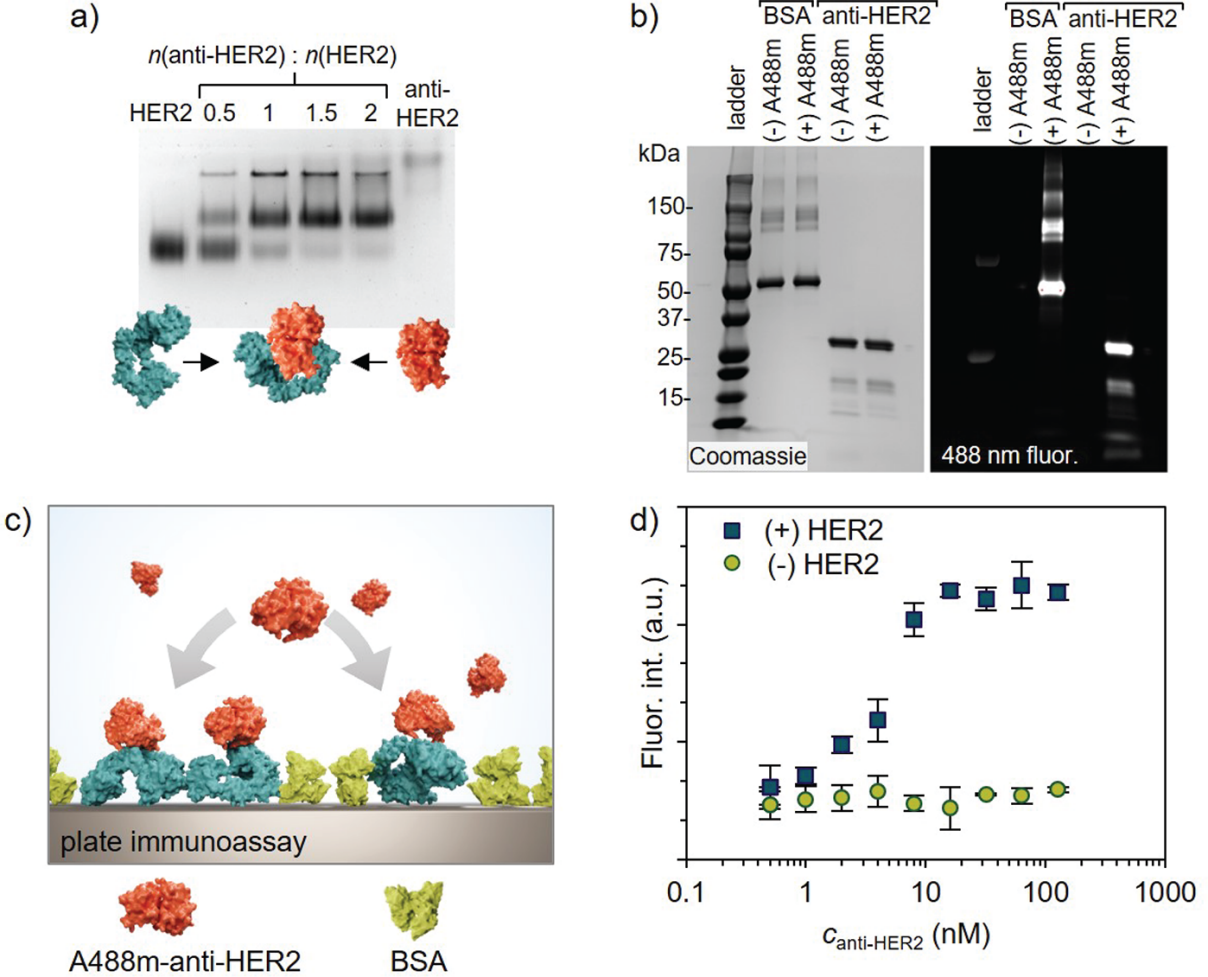
Antibody–antigen interaction for the fluorescence-based plate immunoassay setup. (a) Native polyacrylamide gel electrophoresis (PAGE) shows the interaction between HER2 antigen (concentration 1.8 μM) and anti-HER2 antibody in solution as the molar ratio of antibody:antigen is varied. (b) Comparison of fluorescence signal (A488m, right) and Coomassie blue stained (left) sodium dodecyl sulfate (SDS) PAGE indicating a successful labeling procedure. (c) Assay setup for investigating the interaction between free A488-labeled anti-HER2 (A488m-anti-HER2, orange) and immobilized HER2 (cyan). BSA (green) is used as a blocking agent to avoid unspecific interaction with the uncoated assay plate. (d) Comparison of fluorescence intensity of wells containing HER2 ((+)HER2, blue) or lacking HER2 ((−)HER2, green) after incubation with A488m-anti-HER2. Measurements were performed using triplicate samples, and the averaged results with standard deviation are presented.

In order to set up a fluorescence-based plate immunoassay for DNA origami, native polyacrylamide gel electrophoresis (PAGE) was used to study the interaction between the intact antibody fragment and its corresponding antigen, HER2 (ECD) with a size of 71 kDa. To this end, samples containing either the anti-HER2 only, the HER2 only, or both proteins with a molar excess of 0.5–2× of anti-HER2 were incubated for 1 h at 37 °C (see Figure 2a). Although the molecular weight of HER2 is roughly 2.5 times larger than the molecular weight of anti-HER2, it migrates faster in native conditions (lanes 1 and 6). Upon increase of the anti-HER2 concentration, a gradual disappearance of the HER2 band can be observed, simultaneously with the appearance of a new intermediate band, clearly indicating complex formation between HER2 and anti-HER2. Analysis of the band intensity allows for the determination of the dissociation constant (*K*_d_ = 86 nM; see Note S3 in the Supporting Information).

Having the interaction between antigen and antibody confirmed, a fluorescence assay was established. In a first step, the binding properties of free antibody to immobilized HER2 were investigated by labeling anti-HER2 with a fluorescence signal. This was achieved by the formation of a cysteine–maleimide bond between the free cysteine residue of the antibody fragment and an *N*-maleimide group on the ATTO488-maleimide dye molecule (A488m). Excess dye was removed by spin-filtration, and the concentration of protein and free dye molecules was determined by ultraviolet–visible spectroscopy (UV–vis) absorbance. The outcome of the conjugation reaction was monitored by sodium dodecyl sulfate (SDS) PAGE by comparing the Coomassie blue channel, showing the entire protein content of the sample, and the 488 nm fluorescence channel, visualizing A488m-conjugated proteins only (see Figure 2b). The conjugation reaction was performed for both proteins; BSA is shown in lanes 2–3, and anti-HER2 in lanes 4–5. The migration speed of the denatured proteins does not change upon fluorescence label attachment (Coomassie blue channel, left), but after conjugation a clear signal from the fluorescence channel (right) is obtained only for A488m-containing samples, indicating a successful reaction.

For the plate assay, HER2 (cyan) was immobilized on assay plates by overnight incubation in 50 mM sodium carbonate buffer at 4 °C, followed by incubation with BSA (green) to avoid unspecific binding to the plate. Finally, A488m-anti-HER2 (orange) was incubated, and unbound proteins were removed in a washing step before fluorescence measurement (see Figure 2c). The binding properties were studied using 2 μg mL^–1^ HER2 for coating the wells with the antigen. This concentration was observed to be sufficient for saturating the wells with the antigen for maximal antibody–antigen interaction (see Figure S4). By incubating the wells with A488m-anti-HER2 concentrations ranging from 0.5–125 nM, a significant increase in the fluorescence signal can be observed for HER2 coated wells ((+)HER2, blue) with increasing A488m-anti-HER2 concentration (see Figure 2d).

### Interaction between Bound Antibody and Immobilized Antigen

**Figure 3:**
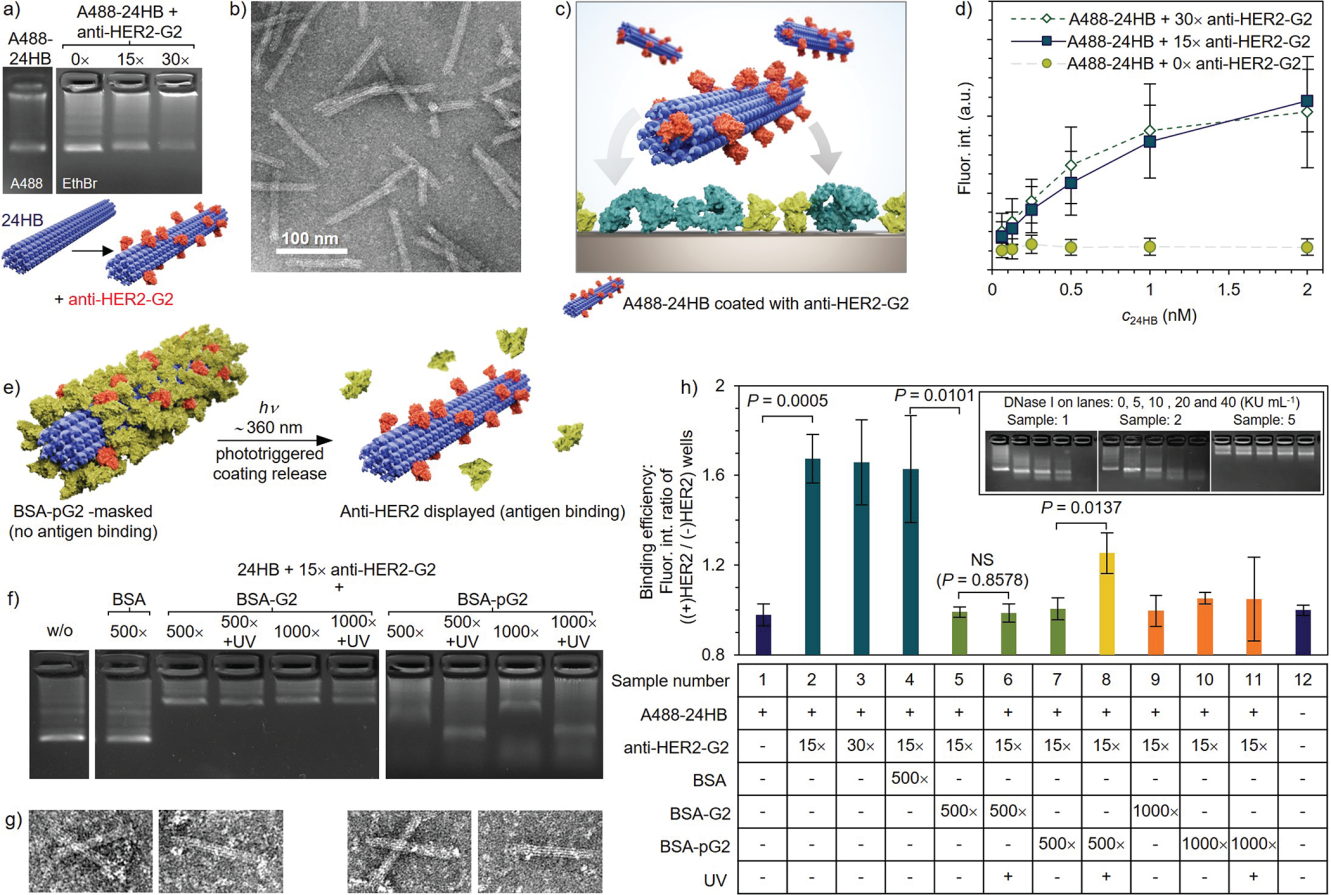
Protein-dendron conjugate complexation with 24HB and investigation of the binding properties of the complex in the plate assay. (a) Electrophoretic mobility shift assay (EMSA) shows that the 24HB band shifts upon complexation with an increasing molar excess of anti-HER2-G2. (b) Transmission electron microscope (TEM) image of 24HB complexed with a 30× excess of anti-HER2-G2. (c) Schematic of the plate-based immunoassay for A488-labeled 24HB which is incubated on plates covered with HER2 (cyan) and BSA (green). (d) Binding properties of the 24HB and anti-HER2-G2 complexes (15× (blue) and 30× (green) excess) to HER2. Uncoated 24HB (olive) serves as a control. The measurements were performed as triplicates and are plotted as the average value with the standard deviation. (e) Schematic of the effect of irradiation with UV-A light at 365 nm on the photolabile BSA coating. BSA dissociates from 24HB and allows the display of anti-HER2. (f) EMSA of 24HB confirms complexation with the BSA components and dissociation upon a 5 min irradiation of different samples with UV-A light. (g) TEM images of 24HB complexed with a 15× excess of anti-HER2-G2 and a 500× molar excess of BSA-pG2 before (left) and after (right) UV irradiation. The image width corresponds to 150 nm. (h) Normalized fluorescence intensities ((+)HER2:(−)HER2 wells) for binding studies in the plate assay before and after UV-A irradiation. Triplicates of the measurement were performed which are presented as the average value with standard deviation. Stability of coated structures against DNase I (inset): 24HB (left), anti-HER2-G2 coated origami (middle), BSA-G2 coated origami (right).

After the assay was established for free anti-HER2, it needed to be tested for DNA nanostructures. To this end, the two-component coating was applied to the DNA origami structure. First, the DNA origami was incubated at room temperature with a molar excess of anti-HER2-G2 (see Figure 3a) ranging from 0 to 30×, and the interaction between the two materials was investigated by monitoring the change in electrophoretic mobility during agarose gel electrophoresis (AGE) and by transmission electron microscopy (TEM) (see Figure 3b). At 15× molar excess an optimal complexation was obtained, since the electrophoretic mobility was not yet visibly changed during AGE. In contrast, at >30× excess of the antibody, slight aggregation of the DNA origami in the gel pocket could be observed. Auvinen et al. described a similar aggregation behavior for hydrophobin-G2 conjugates,^48^ indicating that in both cases the small size of the protein is likely to be the reason for aggregation.

The desired attachment of anti-HER2-G2 to the surface of 24HB was confirmed by studying the binding properties of both, plain 24HB and 15× and 30× molar excess. Briefly, the anti-HER2-G2 complexed 24HB was incubated for 1 h at 37 °C in wells which have been coated with HER2 (cyan) and blocked with BSA (green) (see Figure 3c). In the absence of anti-HER2-G2 the DNA origami does not bind to both HER2 coated and “empty” wells which only contained BSA, suggesting no affinity toward either the antigen or the blocking agent BSA (see Figure 3d). In contrast, increasing 24HB concentration when complexed with either 15× or 30× molar excess of anti-HER2-G2 resulted in an increase in the fluorescence signal. This indicates a successful binding of the antibody on the DNA origami surface as well as the antibodies’ binding ability to the antigen in order to immobilize the 24HB in the wells. Similar binding results are obtained for both tested excess ratios, suggesting that the increase of anti-HER2-G2 molecules does not increase the binding efficiency. This further confirms that the 15× molar excess in the complexation reaction is sufficient for functionalizing the 24HB structure for the required targeting purpose.

### Photoreversibility

Photoreversibility is introduced by adding the second, photolabile coating compound, BSA-pG2, to the system. After incubating the anti-HER2-G2 complexed 24HB with an excess of BSA-pG2, the electrophoretic mobility was monitored to confirm complexation. A steady decrease in the mobility is observed with increasing BSA-pG2 excess without any aggregation in the well even though up to 2,500× molar excess was used (see Figure S5). Similar behavior of BSA-G2 coated DNA origami structures was also observed by Auvinen et al. For further experiments, 500× and 1,000× excesses were chosen.

To demonstrate the photoreversibility, the double-coated samples were irradiated with UV-A light (4 × 15 W lamps) at a wavelength of λ = 365 nm, which triggers the cleavage of the photolabile *o*-nitrobenzyl group and subsequent dissociation of the BSA from the DNA origami structure and display of the antibody fragment (see Figure 3e). Testing irradiation for 1 to 10 min (see Figure S6) resulted in partial release of the DNA origami from BSA after 1 min and full release after 3–5 min. To ensure full dissociation of BSA, samples tested in the fluorescence assay were irradiated for 5 min.

The responsiveness to UV irradiation is exclusive for BSA-pG2 coated structures; bare origami structures or 24HB coated with BSA-G2 is not affected (see Figure 3f, lanes 4 and 6). Bare origami (Figure 3f, lane 1) is used as reference for monitoring the electrophoretic mobility; in Figure 3f all samples tested in the fluorescence assay are shown. While both BSA-pG2 and BSA-G2 coated samples show a significant decrease in the mobility, it is notable that the sample is not affected by free BSA (lane 2). An increase in the electrophoretic mobility (see Figure 3f, lanes 7–10, and Figure S6) suggests a full release from BSA between 3 and 5 min. Possible structural defects due to transfer of the DNA origami into an environment with altered salt concentration upon complexation (see Figure 3g, left) and UV-A irradiation (see Figure 3g, right) could not be detected in TEM. This is in line with reports from Chen et al., who could not detect visible defects even upon high-dose UV-A irradiation.^56^

The binding properties of samples with different coatings were studied based on their fluorescence intensities in the established assay (see Figure 3h). The measurements were performed as triplicates, and each sample was incubated in both (+)HER2 and (−)HER2 wells. To exclude an unspecific binding effect, the ratio between these two fluorescence intensities was plotted. As soon as BSA is added to the system, in the form of either BSA-G2 (samples 5 and 9) or BSA-pG2 (samples 7 and 10), the fluorescence signal is in the range of plain DNA origami (sample 1) and the negative control (sample 12), suggesting that the antibody–antigen interaction is prohibited. However, as soon as the samples are irradiated, the signal of the BSA-pG2 coated sample (500× , sample 8) is significantly increased, while the BSA is not released from the BSA-G2 coating (sample 6), which is in line with the results from AGE. A first analysis of the data suggests that although a full release of the DNA origami from BSA-pG2 is observed in AGE, the maximum binding capability in the assay is not fully recovered. This could be caused by incomplete photoinduced release that is not measurable by AGE as well as due to the competitive binding behavior of BSA-pG2 and anti-HER2-G2 to the origami structure. A large excess of BSA-pG2 might result in dissociation of anti-HER2-G2 from the structure. Additionally, free BSA in the solution might have an influence on binding, as indicated in sample 4. A decrease in the fluorescence intensity is observable, which is enhanced with increasing concentration of free BSA. Free BSA arises after UV irradiation due to photocleavage and can also originate from residuals of the conjugation reaction, which were not fully removed during purification. Taking the free BSA and its decrease in maximum binding capability into account, full recovery of the binding properties is obtained, especially for low BSA-pG2 coating ratios (see Figure S7). It is notable that we observe a reversible “camouflaging” effect caused by adding a BSA protein corona to the anti-HER2 complexed DNA origami. A similar effect has been described by Salvati et al., who reported a loss of the targeting properties of silica nanoparticles in complex biological media.^57^ In our system, the reversibility is significant for low molar excesses of BSA-pG2; however, for structures coated with larger excesses, it is hardly detectable (sample 11), likely due to the competitive nature of the two components as described above. We, furthermore, noticed an influence of free BSA on the binding properties which does not have any effect on the response to the stimuli, as shown with AGE, but might limit the application for high BSA-pG2 coating ratios or in the presence of protein-rich solutions. However, performing the assay in cell medium (Dulbecco’s Modified Eagle Medium (DMEM) supplemented with a final concentration of 10% fetal bovine serum (FBS)) still showed significant binding interaction between the anti-HER2-G2 complexed origami structures and HER2 (see Figure S8). Alternative purification methods for both conjugation and complexation reactions could improve the removal of free compounds and allow system refinement. Additionally, fine-tuning of the complexation steps including the presence of free anti-HER2-G2 upon BSA-pG2 addition or covalent attachment of anti-HER2 to the DNA nanostructure could be a promising option to further improve the system’s reversibility.

Additionally, the DNA structures were found to be better protected against DNase I digestion when the BSA coating is applied (Figure 3h, right inset), while the effect for the anti-HER2-G2 complexed samples is negligible (Figure 3h, middle inset) compared to plain 24HB (Figure 3h, left inset) which is in line with the results from Auvinen et al.^48^

## Conclusion

In conclusion, we have successfully developed a facile, modular, stimuli-responsive, two-component coating system for DNA origami nanostructures purely based on electrostatic interactions. By the stepwise addition of components with different molecular weights, we have observed a “camouflaging effect” of the second, bulkier component, BSA, toward the antibody fragment. However, due to the responsiveness toward UV-A light, the BSA coating could be fully removed, and antibody binding properties were restored. The effect was investigated by monitoring the binding properties of DNA origami to the HER2 antigen in a fluorescence-based plate immunoassay. Additionally, BSA enhances the DNA nanostructures’ stability against DNase I.

Our coating approach is highly versatile, since any protein with a free cysteine residue can be attached to the dendron, allowing for further tuning of the nanostructure’s properties, for instance toward a/several specific target(s) by turning it into a multicomponent system. Due to the high addressability of the nanostructures, not even all of them have to be electrostatically attached; for example, drugs/therapeutic proteins could be hybridized on the structure and would still be protected by the bulky primary coating. Together with its responsiveness to environmental stimuli, such as electromagnetic radiation, it can be considered attractive for application in various fields.

## Experimental Section

### Folding and Purification of DNA Origami

The 24HB nanostructure (scaffold purchased from Tilibit Nanosystems, staples from Integrated DNA Technologies) was folded in a one-pot reaction using a Whatman Biometra TGradient Thermocycler. For the attachment of the fluorescence molecules, 12 strands per each end were exchanged with staples containing overhangs at the 3′-ends (see Note S9). Briefly, 100 nM of a 7560 nt long scaffold were mixed with 500 nM of each staple strand and annealed using a thermal ramp (cooling from 65 to 59 °C with a ramp of −4.0 °C h^–1^ and from 59 to 40 °C with a ramp of −0.33 °C h^–1^). The reaction was performed in a buffered environment (“folding buffer (FOB)”, 1× Tris-Acetate-EDTA (1× TAE) buffer containing 40 mM Tris-acetate and 1 mM EDTA and 17.5 mM MgCl_2_). The folded structures were purified from excess staple strands using polyethylene glycol (PEG) precipitation, as previously reported by Stahl et al.^58^ 200 μL of ∼20 nM DNA origami solution were 4-fold diluted with FOB to ∼5 nM and mixed in a 1:1 ratio with PEG precipitation buffer (1× TAE, 505 mM NaCl, 15% (w/v) PEG8000), followed by a centrifugation step at 14,000*g* for 30 min. After careful removal of the supernatant, the pelleted origami structures were resuspended in a 1× initial volume of the FOB and incubated overnight at 30 °C and 600 rpm in an Eppendorf ThermoMixer C. The strands with fluorescent dyes (Integrated DNA Technologies) were added in 10× molar excess per attachment site and annealed using a thermal ramp (cooling from 40 to 20 °C with a ramp of −0.1 °C h^–1^). Excess fluorophore-labeled strands were removed by PEG precipitation as described above. The DNA origami concentration was estimated by measuring the absorbance of the sample at 260 nm (*A*_260_) using a BioTek Eon Microplate spectrophotometer (2 μL sample volume, Take3 plate). ε was estimated to be to be 1.076 × 10^–8^ M^–1^ cm^–1^ based on the number of nonhybridized and hybridized nts in the structure.^59^

### Agarose Gel Electrophoresis

Agarose gel electrophoresis was used to monitor the folding of 24HB as well as the removal of excess staples. Furthermore, the binding behavior of the proteins to 24HB and the progression of DNase I digestion were studied using electrophoretic mobility shift assay (EMSA). To this end, a 2% (w/v) agarose gel was prepared in 1× TAE buffer, supplemented with 11 mM MgCl_2_ and ethidium bromide (EtBr, final concentration 0.46 μg mL^–1^) for visualization of the DNA. The samples (volumes ranging from 10–24 μL) were diluted in 6× gel loading dye solution before addition onto the gel, which was run at 90 V for 45 min in 1× TAE, 11 mM MgCl_2_ buffer. The results were imaged under either UV light (EtBr channel) or blue light (Alexa488 channel) using a ChemiDoc MP system (Bio-Rad).

### Transmission Electron Microscopy

The samples were prepared by deposition of the DNA origami solution on a plasma cleaned (15 s, NanoClean 1070, Fischione Instruments) Formvar carbon coated copper grid (FCF-400Cu, Electron Microscopy Sciences) similar to the protocol reported by Castro et al.^31^ Samples with a concentration of 4 nM (3 μL) were incubated for 3 min while samples with 2 nM (5 μL) concentration were incubated for 3.5 min before blotting against filter paper and subsequent negative staining in aqueous 2% (w/v) uranyl formate solution (pH adjusted by addition of 25 mM NaOH). Staining was performed by immersing the grid in a 5 μL stain droplet which was immediately removed followed by a 45 s incubation after immersion in a 20 μL droplet. After blotting away the excess, the samples were dried for at least 20 min before imaging with a FEI Tecnai 12 Bio-Twin microscope at an acceleration voltage of 120 V. For samples containing BSA, an additional washing step was added before the staining procedure by immersing the grid in 10 μL of complexation buffer (0.16× TAE, 2.8 mM MgCl_2_, 0.8 mM 4-(2-hydroxyethyl)-1-piperazineethanesulfonic acid (HEPES), 0.2× phosphate buffered saline (PBS) salts, and 150 mM NaCl) for 10 s.

The difference in sample concentration was caused by the removal of free BSA in the coated samples by spin-filtration. To this end, 100 kDa molecular weight cut off (MWCO) spin-filters (Amicon Ultra, Merck, 0.5 mL) were washed with 400 μL of complexation buffer by centrifugation at 14,000*g* for 5 min. 10 μL of the sample was mixed with 190 μL of the complexation buffer and centrifuged for 10 min at 6,000*g*. Under thorough mixing, 200 μL of the complexation buffer was added, and the solution was centrifuged for 10 min at 6,000*g*. This step was repeated three times before the sample was collected into a fresh tube by inverting the filter and centrifugation at 1,000*g* for 2.5 min.

### Preparation of Anti-HER2

The anti-HER2 was recombinantly expressed in *Escherichia coli* RV308 strain, adapted from a published protocol.^51^ Briefly, a single colony was used to inoculate a starting culture (16 mL, lysogeny broth medium supplemented with 1% (v/v) glucose and 400 μg mL^–1^ ampicillin) which was incubated overnight at 37 °C, 220 rpm. The main culture (400 mL, terrific broth medium with 100 μg mL^–1^ ampicillin) was inoculated with 2% (v/v) of the starting culture (8 mL) and incubated at 37 °C at 180 rpm until the optical density at 600 nm (OD_600_) reached 4.0–5.0. Protein expression (16–20 h at 30 °C, 180 rpm) was induced with isopropyl-β-d-thiogalactoside (IPTG, 1 mM final concentration). The cells were harvested by a 15 min centrifugation at 13,700*g*.

For the purification of the protein containing a *C*-terminal His-Tag from the medium, His-beads (HisPur Ni-NTA resin, ThermoScientific) were used. To this end, the medium was diluted 1:1 into equilibration buffer (20 mM sodium phosphate, 300 mM NaCl, 10 mM imidazole, pH 7.4) and incubated with the His-beads (2.5 mL of resin was sufficient for 170 mL of medium, prepared according to the manufacturer’s instructions) on an end-over-end shaker for 1 h. The His-beads were sedimented by centrifugation for 2 min at 700*g* and subsequently washed 6 times by addition of two resin-bed volumes (5 mL) of washing buffer (20 mM sodium phosphate, 300 mM NaCl, 25 mM imidazol, pH 7.4) and centrifugation for 2 min at 700*g*. The bound protein was eluted by thoroughly mixing the beads with one resin-bed volume (2.5 mL) of elution buffer (20 mM sodium phosphate, 300 mM NaCl, 250 mM imidazole, pH 7.4) and a centrifugation step for 2 min at 700*g*. The eluted fractions were pooled and filtered with a 0.45 μm syringe filter before upconcentration (10 kDa MWCO PES, Vivaspin 20, Sartorius, 20 mL; centrifugation for 10 min at 3,500*g*). Dithiothreitol (DTT, final concentration 2 mM) was added to reduce the C-terminal free cysteine residue of anti-HER2. After incubation for 30 min at 37 °C, the protein was loaded onto a desalting column (HiTrap Desalting, Cytiva; 2 × 5 mL volume) and eluted in 1× PBS containing 1 mM EDTA, pH 6. Fractions with absorbance at 280 nm were pooled and if necessary further upconcentrated by spin-filtration (centrifugation for 10 min at 3,500*g*). All centrifugation steps were performed at 4 °C.

The concentration was determined using Lambert–Beer’s law with ε_280_ = 50,100 M^–1^ cm^–1^. The overall amount of proteins present during the expression and purification was analyzed with an SDS-PAGE (4–20%, Mini-Protean TGX precast polyacrylamide gel, BioRad). The gel was run in running buffer containing 25 mM Tris, 192 mM glycine, 0.1% SDS for 30 min at 200 V and imaged using a ChemiDoc MP gel imaging system (Bio-Rad) after Coomassie blue staining.

### Protein Conjugation with Dendron Structures

The conjugation of the dendrimers to the proteins was achieved by cysteine-maleimide coupling. BSA was dissolved in deionized water and mixed with in deionized water resuspended (photolabile) dendron in 5× molar excess. The conjugation reaction was performed in 86 mM sodium phosphate buffer containing 16 mM EDTA for 36 h. Unconjugated pG2 was removed by spin-filtration (Amicon Ultra, 0.5 mL, 10 kDa MWCO). After washing the filter with 400 μL 1× PBS (5 min, 14,000*g*), 100 μL of the BSA-pG2 conjugation reaction mixture was added into the filter together with 400 μL 1× PBS. After centrifugation (10 min, 14,000*g*), 350 μL of 1× PBS was added and the centrifugation repeated. The PBS wash was performed 3 times, before the filter was inverted for sample elution. The concentration was estimated based on the absorption at 380 nm measured with a BioTek Eon Microplate spectrophotometer (2 μL sample volume, Take3 plate).

Unreacted components from BSA-G2 samples were removed by fast protein liquid chromatography (FPLC) using a HiTrap Heparin column (5 mL, Cytiva). The conjugate was eluted by increasing the NaCl concentration (20 mM sodium phosphate buffer, 0–2 M NaCl, pH 6.7). The conjugate concentration was estimated using Lambert–Beer’s law by measuring the absorbance at 280 nm (ε_280_ = 43,824 M^–1^cm^–1^).

G2 was conjugated to anti-HER2 with 2× molar excess in a 1× PBS pH 7 buffered environment. The mixture was incubated for 2 h at room temperature on an end-to-end shaker before transfer to 4 °C for ∼40 h. Unconjugated components were removed by increasing the NaCl concentration (0–2 M in 20 mM HEPES buffer containing 20 mM EDTA, pH 7) when eluting from a HiTrap Heparin column (5 mL). Eluted fractions of anti-HER2-G2 were pooled and upconcentrated (10 kDa MWCO; 10 min, 3,200*g*) before dialysis (10 kDa MWCO dialysis cups, Slize-A-lyzer, ThermoFisher) against 10 mM HEPES pH 7 at 4 °C to remove the NaCl.

### Protein Conjugation with Fluorophore

To ensure a reduced state of the C-terminal cysteine residue of anti-HER2 before conjugation, the protein was incubated for 30 min with a 100× molar excess of tris(2-carboxyethyl)phosphine (TCEP). Simultaneously with the removal of TCEP, a buffer exchange to 1× PBS pH 7 was performed by spin-filtration (Amicon Ultra 10 kDa MWCO; 10 min 14,000 *g*). Subsequently, the protein was mixed with ATTO488-maleimide (ATTO-TEC, in 1× PBS pH 7 containing 10% (v/v) dimethylformamide) which was in 6.4× molecular excess. After overnight incubation at 30 °C and 400 rpm, unreacted A488 maleimide was removed by 6 rounds of spin-filtration (Amicon Ultra, 10 kDa MWCO; 8 min, 14,000*g*). The outcome of the conjugation reaction was analyzed with an SDS-PAGE (4–20% Mini-Protean TGX precast polyacrylamide gel, BioRad) which was run for 30 min at 200 V in a running buffer containing 25 mM Tris, 192 mM glycine, and 0.1% SDS. After imaging the A488 fluorescence using the Alexa488 channel of a ChemiDoc MP gel imaging system (Bio-Rad), the gel was stained with Coomassie and reimaged.

### Interaction of HER2 and Anti-HER2 in Solution

Native PAGE (separation gel 8% acrylamide/bis(acrylamide) (29:1) in 0.3 M Tris pH 8.8; stacking gel 4% acrylamide/bis(acrylamide) (29:1) in 0.3 M Tris pH 6.8) was used for studying the interaction between anti-HER2 and the HER2 ECD in solution. The antibody was mixed with the antigen in molar ratios ranging from 0–2× excess and incubated for 60 min at 37 °C. The samples were diluted into 2× native PAGE sample buffer (62.5 mM Tris, 40% (v/v) glycerol, 0.01% (w/v) bromophenol blue) upon loading onto the gel. Separation was achieved by running the gel for 55 min at 200 V in 25 mM Tris, 192 mM glycine pH 8.3. After Coomassie staining, the gel was imaged with a ChemiDoc MP gel imaging system (Bio-Rad).

### Complexation of DNA Origami and Protein Conjugates

The complexation of DNA origami and protein conjugates was performed in two steps. First, the PEG-purified origami structure in 1× FOB was mixed with the desired excess of anti-HER2-G2 in 10 mM HEPES buffer. The mixture was diluted in deionized water to a final DNA origami concentration of 4 nM and the NaCl concentration adjusted to 150 mM. The samples were incubated for 20–30 min at 300 rpm before BSA-(p)G2 in 1× PBS containing 150 mM NaCl was added. The incubation was continued for 20–30 min at 300 rpm. Finally, the solution contained 3.2 nM DNA origami in 0.16× folding buffer (0.16× TAE, 2.8 mM MgCl_2_, 0.8 mM HEPES, 0.2× PBS containing 150 mM NaCl). The outcome of the complexation was analyzed using AGE.

### Photoreversibility

For the removal of the BSA coating, a 10 μL droplet was placed into a UV-reactor made of four 15 W UV lamps (λ = 365 nm, Nasc). The samples were irradiated for 5 min. Due to evaporation, the volume of the samples was adjusted with deionized water to the original sample volume to ensure uniform DNA origami concentration when comparing samples on an agarose gel.

### Plate Assay

The binding properties of anti-HER2 were investigated in a plate-based immunoassay by monitoring the fluorescence intensity of the ATTO488-labeled antibody fragment and the ATTO488-labeled 24HB (A488). For the final assay, the HER2 protein (ECD, Sino Biological) was diluted to 2 μg mL^–1^ in 50 mM sodium carbonate buffer pH 9.6. Immobilization of the protein on black 96-well MaxiSorp immunoplates (ThermoFisher) was achieved by incubation of 100 μL solution per well overnight at 4 °C. The wells were washed 4 times with washing buffer containing 1× PBS, 200 mM NaCl, and 0.05% Tween20 (pH 7.4) and blocked with 1% (w/v) BSA in 1× PBS, 0.05% Tween20 (pH 7.4) for 1–2.5 h at room temperature. After washing 4 times with washing buffer and once with 1× PBS (pH 7.4), 100 μL of the sample was added to the wells and incubated for 37 °C for 1 h.

The samples were complexed in the same conditions as described above; however, the HEPES concentration was decreased to 1.2 mM. For the plate assay, a sample volume of 220 μL with a DNA origami concentration of 1 nM was used. To this end, the 3.2 nM samples were diluted to 0.1× TAE, 1.75 mM MgCl_2_, 0.75 mM HEPES, 1× PBS containing 150 mM NaCl. Before the fluorescence measurement, the wells were washed 3 times with wash buffer and once in 1× PBS (pH 7.4). The measurement was performed in 1× PBS (pH 7.4). To this end, fluorescence spectra were collected from 480 nm excitation using a BioTek Synergy H1 microplate reader, and the spectra were integrated between 520 and 570 nm to obtain the final fluorescence intensities.

The fluorescence intensities from three separate experiments were averaged. To investigate the influence of unspecific binding, negative controls were implemented by preparing the wells without adding the HER2 protein. The ratio of the fluorescence intensities is presented in the Results and Discussion section. To evaluate the significance (P) of the intensity change, an unpaired *t*-test was performed.

### Digestion Assays with DNase I

To study the stability against DNase I, 1 μL of DNase I (stock concentrations varying from 0 to 560 KU mL^–1^) was added to 12 μL of the sample and 1 μL of 14 mM CaCl_2_, resulting in buffer conditions of 0.4 mM HEPES, 0.2× PBS containing 150 mM NaCl, 2.8 mM MgCl_2_, 1 mM CaCl_2_. The DNA nanostructures were digested for 60 min at 37 °C. Before pipetting the samples onto an agarose gel, the coating was removed by addition of heparin, a competitive negatively charged agent. A 50× molar excess of heparin sodium salt dissolved in water (in a volume of 6 μL) was sufficient to disassemble the protein-dendron coating within 5 min.

## Supporting Information Available

Folding of the 24 helix bundle origami structure; cation exchange chromatogram of BSA-G2; dissociation constant determination of anti-HER2 by PAGE; comparison of different HER2 concentrations in an A488m-anti-HER2 plate assay; complexation of BSA-pG2 and anti-HER2-G2 coated 24HB origami; time-dependent release of 24HB upon UV irradiation; free BSA normalized plate assay results; plate assay results in cell medium; and 24HB design and staple sequences (PDF)
