# Supplementary material for: Optically Responsive Protein Coating of DNA Origami for Triggered Antigen Targeting
Source: ACS Appl Mater Interfaces. 2022 Aug 19;14(34):38515–24. doi: 10.1021/acsami.2c10058 (PMC9437894; doi:10.1021/acsami.2c10058)
Supplement: Supplementary file 1 — am2c10058_si_001.pdf [file am2c10058_si_001.pdf]

Supporting Information for Optically Responsive Protein Coating of DNA Origami for Triggered Antigen Targeting

Iris Seitz[1], Heini Ijäs[1,2], Veikko Linko[1,3*] & Mauri A. Kostiainen[1,3*]

[1]Biohybrid Materials, Department of Bioproducts and Biosystems, Aalto University, P.O. Box 16100, 00076 Aalto, Finland

[2]Ludwig-Maximilians-University, Geschwister-Scholl-Platz 1, 80539 Munich, Germany

[3]LIBER Center of Excellence, Aalto University, P.O. Box 16100, 00076 Aalto, Finland

[*]Correspondence and requests for materials should be addressed to veikko.linko@aalto.fi or mauri.kostiainen@aalto.fi

Contents

	Page
Note S1: Folding of 24 helix-bundle origami structure (Supplementary Figure 1)	S3
Note S2: Cation exchange chromatogram of BSA-G2 (Supplementary Figure 2)	S3
Note S3: Dissociation constant determination of anti-HER2 by PAGE (Supplementary Figure 3)	S3
Note S4: Comparison of different HER2 concentrations in A488m-anti-HER2 plate assay (Supplementary Figure 4)	S4
Note S5: Complexation of BSA-pG2 and anti-HER2-G2 coated 24HB origami (Supplementary Figure 5)	S4
Note S6: Time-dependent release of 24HB upon UV irradiation (Supplementary Figure 6)	S5
Note S7: Free BSA normalized plate assay results (Supplementary Figure 7)	S5
Note S8: Plate assay results in cell medium (Supplementary Figure 8)	S5
Note S9: 24HB design and staple sequences (Supplementary Figures 9–10, Supplementary Table 1)	S6

Note S1: Folding of 24 helix-bundle origami structure

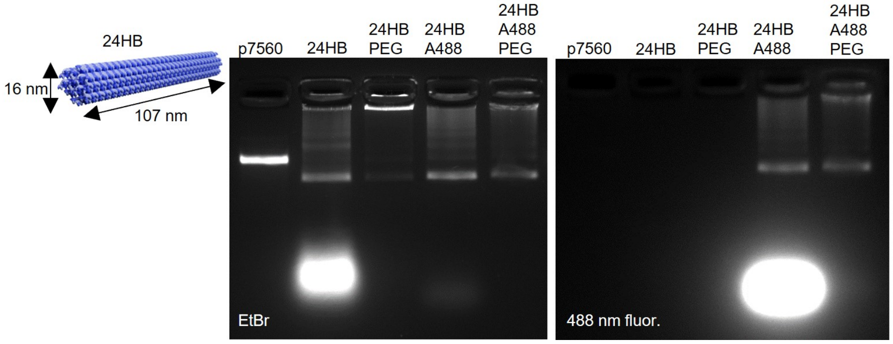

Figure S1. Folding and purification of 24HB origami structure and attachment of A488 strands. The folded 24HB structures migrate faster in AGE than the p7560 scaffold. After PEG purification, the excess of free staple strands is removed and A488 labelled strands are annealed to the structure which can now be monitored in ethidium bromide (EtBr) channel (left) and 488 nm fluorescence channel (right).

Note S2: Cation exchange chromatogram of BSA-G2

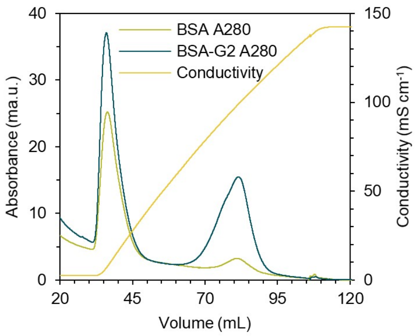

Figure S2. Purification of BSA-G2 from unreacted compounds after the conjugation reaction using a HiTrap Heparin column. The conjugate (blue) is eluted by increasing the NaCl concentration.

Note S3: Dissociation constant determination of anti-HER2 by PAGE

Analysis of the band intensity of HER2 in ImageJ allows plotting the molar fraction of unbound HER2 *vs*. the molar excess of anti-HER2. Fitting with a 1:1 supramolecular binding model results in the following dissociation constant

$$K_\mathrm{d} = \frac{[\text{anti-HER2-scFv}^\mathrm{C}]\ [\text{HER2}]}{[\text{anti-HER2-scFv}^\mathrm{C}\text{-HER2}]} = 86\ \text{nM} \tag{S1}$$

The obtained value is two orders of magnitude higher than previously reported (1). A more reliable value could be obtained by using antibody and antigen concentrations in the pM–nM range instead of µM (2). Furthermore, the buffer conditions might have an influence on the dissociation constant (3).

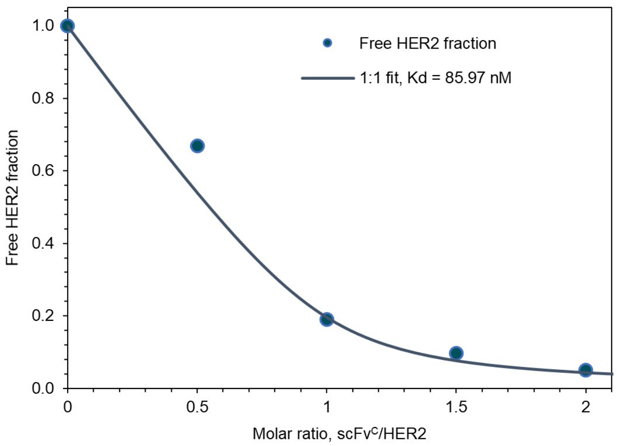

Figure S3. Determination of the K_d value of anti-HER2 based on intensity analysis of the bands shown in Figure 2a by fitting a 1:1 supramolecular binding model.

Note S4: Comparison of different HER2 concentrations in A488m-anti-HER2 plate assay

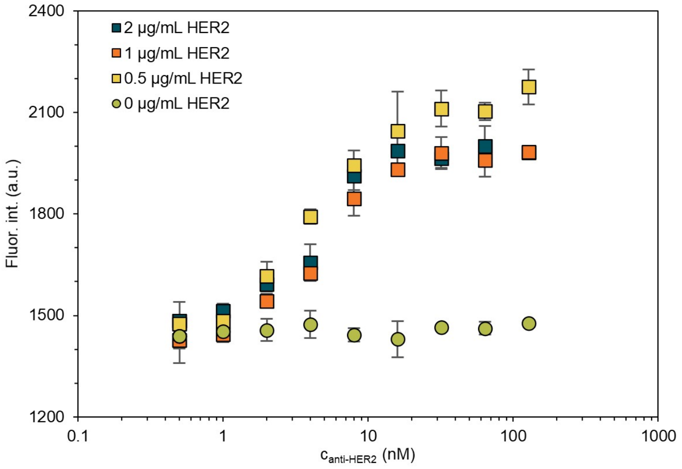

Figure S4. In addition to 0 and 2 µg mL^{-1} HER2 concentration, 0.5 and 1 µg mL^{-1} were tested for their interaction with A488m-anti-HER2 when immobilized on the assay plate. Interestingly, no significant differences in the binding assay can be observed as long as HER2 is present suggesting that the used HER2 concentration is sufficient to saturate the wells. Triplicate samples were used to perform the measurements, and the average value with standard deviation is presented.

Note S5: Complexation of BSA-pG2 and anti-HER2-G2 coated 24HB origami

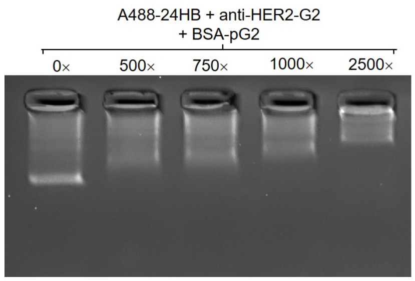

Figure S5. Complexation reaction of anti-HER2-G2 coated 24HB with increasing excess of BSA-pG2. A clear shift in mobility can be observed at a ratio of 500× excess. Further mobility decrease upon addition of up to 2,500× excess suggests gradual saturation of the DNA origami surface with BSA-pG2.

Note S6: Time-dependent release of 24HB upon UV irradiation

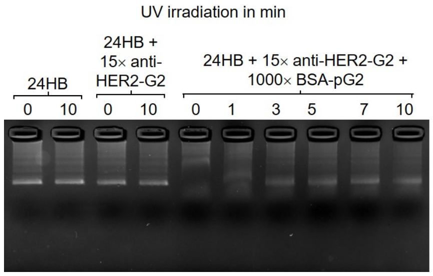

Figure S6. Treatment of BSA-pG2 complexed 24HB structures with UV-A light (λ = 365nm) for up to 10 min shows a time-dependent release process by monitoring the electrophoretic mobility. Full release is observed after 3-5 min where the mobility corresponds to untreated 24 HB. Treatment of plain 24HB or anti-HER2-G2 coated 24HB does not show any response.

Note S7: Free BSA normalized plate assay results

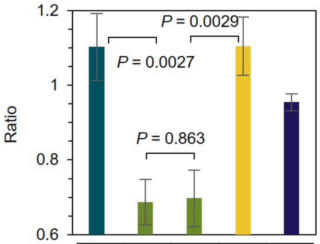

Sample number	2	5	7	8	12
A488-24HB	+	+	+	+	-
anti-HER2-G2	15×	15×	15×	15×	-
BSA	-	-	-	-	-
BSA-G2	-	500×	-	-	-
BSA-pG2	-	-	500×	500×	-
UV	-	-	-	+	-

Figure S7. The plate assay results (Figure 3h) were normalized against free BSA by dividing (+)HER2 to (-)HER2 ratios of two-component coated 24HB and anti-HER2-G2 24HB with corresponding free BSA concentration. Increasing amount of free BSA was found to reduce the maximum binding ability of with anti-HER2-G2 complexed 24HB structures. While the binding properties of BSA coated structures are not affected, full recovery of the binding after UV-A irradiation for 500× excess of BSA-pG2 is observed. The measurements were conducted using triplicate samples and the average value with standard deviation is shown.

Note S8: Plate assay results in cell medium

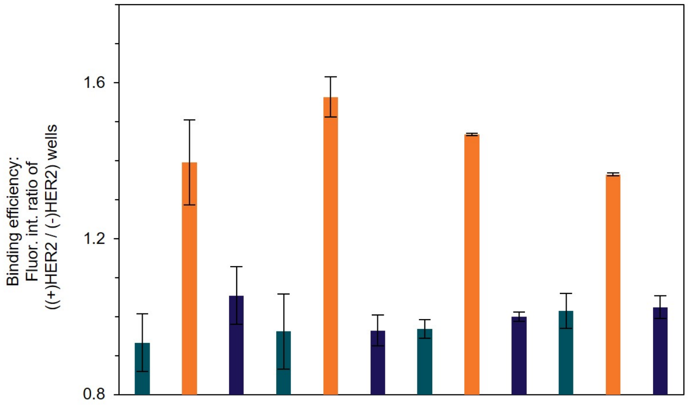

Sample number	1	2	3	4	5	6	7	8	9	10	11	12
A488-24HB	+	+	-	+	+	-	+	+	-	+	+	-
anti-HER2-G2	-	15×	-	-	15×	-	-	15×	-	-	15×	-
DMEM	-	-	-	+	+	+	+	+	+	+	+	+
FBS	-	-	-	-	-	-	10%	10%	10%	20%	20%	20%

Figure S8. Normalized fluorescence intensities ((+)HER2:(-)HER2 wells) for binding studies in the plate assay comparing PBS with cell medium. DMEM was supplemented with a starting concentration of FBS ranging from 0–20 % which resulted in a final concentration of 0–10 % in the binding experiments. The measurements were performed as dublicates and the average and standard deviation are presented.

Note S9: 24HB design and staple sequences

The design of the 24HB structure has previously been described by Ijäs et al. (4), however, 24 staple strands were exchanged to staples containing an overhang for the attachment of A488. The blueprints of the structure are given in Figures S9 and S10. The exchanged staples are highlighted in green. Their sequences are listed in Table S1.

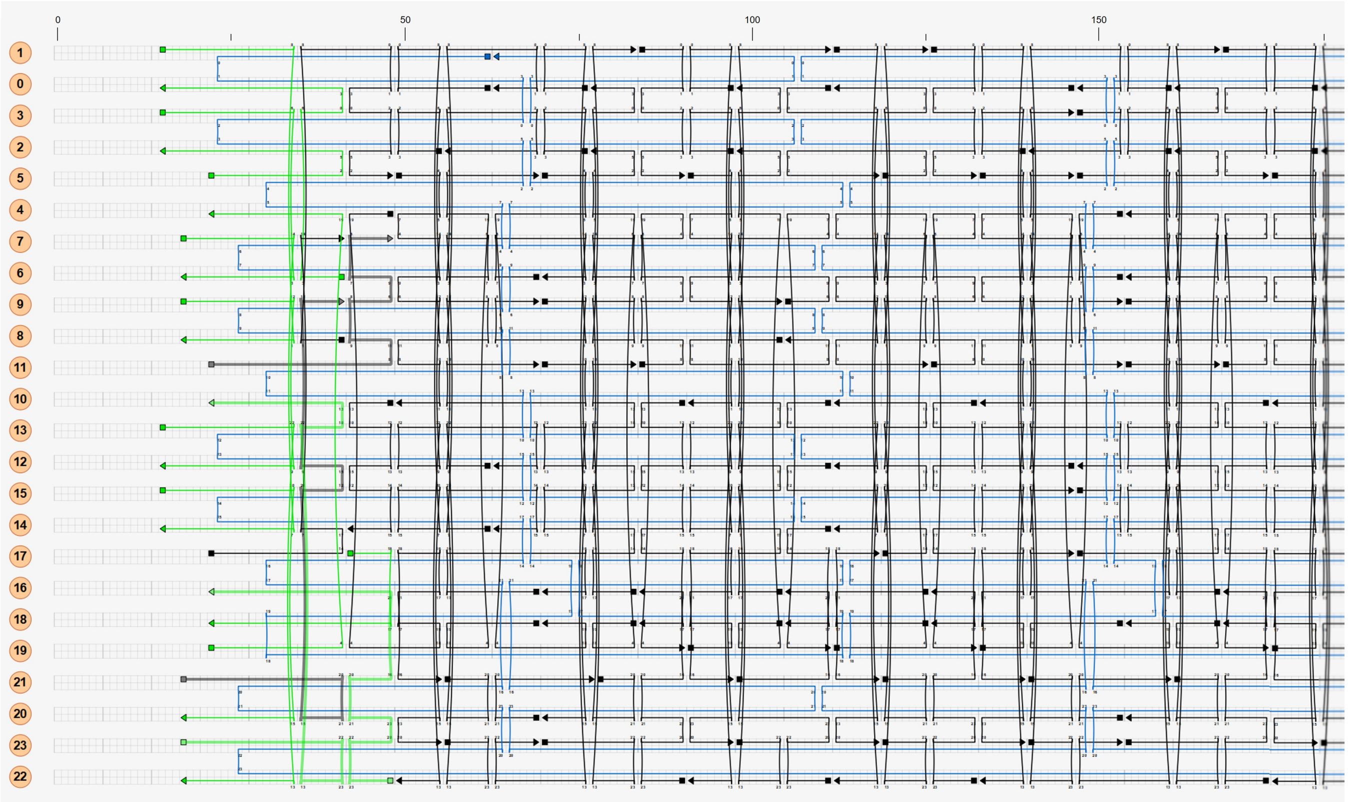

Figure S9. Left part (grid positions 0–175) of the caDNAno blueprint of 24HB. Staples with overhang sequence for annealing to A488-modified strand are highlighted in green, the p7560 scaffold is displayed in blue and the remaining staples in black.

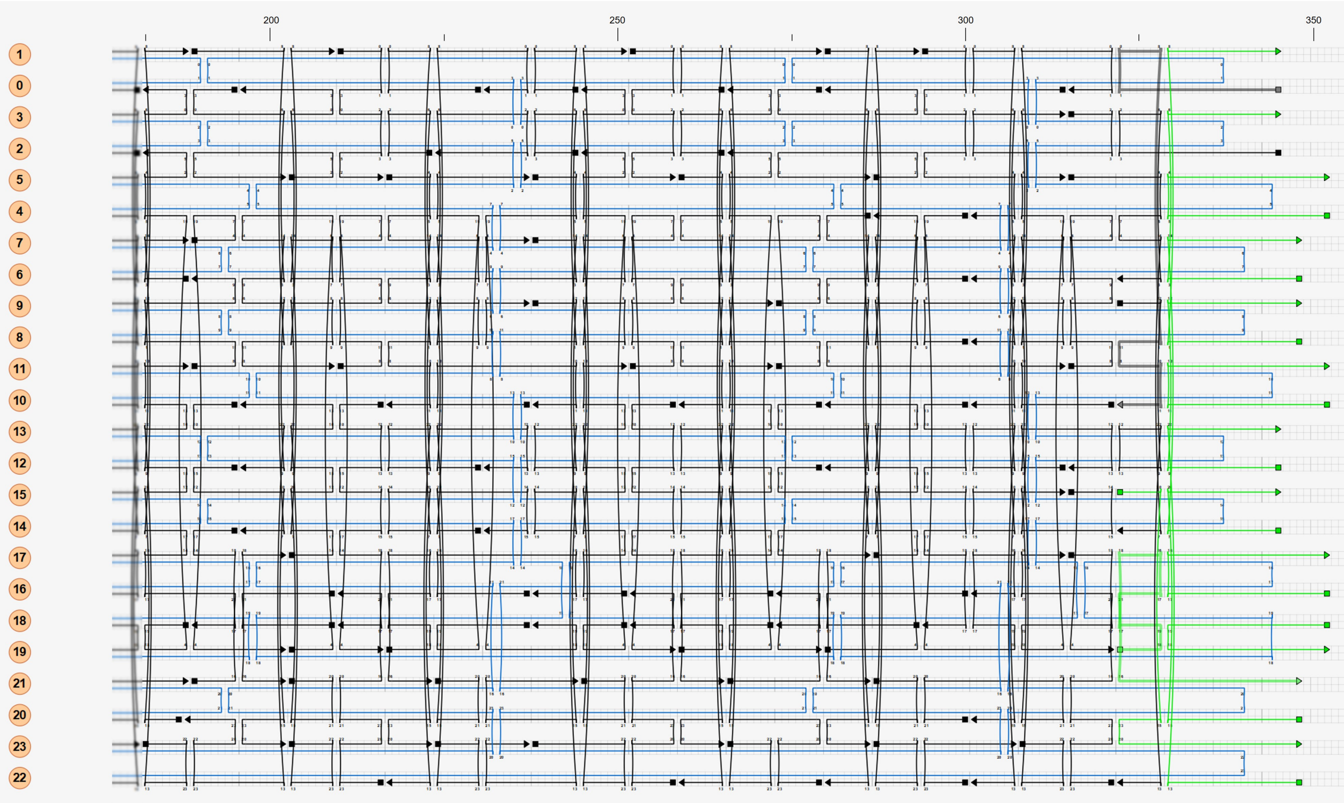

Figure S10. Right part (grid positions 175–352) of the caDNAno blueprint of 24HB. Staples with overhang sequence for annealing to A488-modified strand are highlighted in green, the p7560 scaffold is displayed in blue and the remaining staples in black.

Table S1. Staple strand sequences for attachment of A488-modified strand to 24HB origami. While the nucleotides complementary to the scaffold are written in capital letters, any overhang sequence is indicated by lowercase letters. Note, that the sequence complementary to the A488-modified strand is italicised. Nucleotide bases complementary to the scaffold strand are written in capital letters. The 8× poly-T overhang sequences in staples #174–202 have been indicated by lowercase letters. Start pos. and end pos. indicate the positions of the staple strand termini in the caDNAno design (helix number[grid position]).

#	**Start pos.**	**End pos.**	**Sequence**
r1	8[348]	1[345]	ttttttttGCAAGGATAAAACAATTCTGC*ttttttctcctttccc*
r3	6[348]	3[345]	ttttttttAAGCTAAATCGGAATAACCTG*ttttttctcctttccc*
r5	4[352]	5[352]	ttttttttAGCATTAACATCCAATTTCTACTAATAGTAGT*ttttttctcctttccc*
r7	14[345]	7[348]	ttttttttTCATTGCCTCCTCAGAGCATA*ttttttctcctttccc*
r9	12[345]	9[348]	ttttttttATAAATTAACTTTATTTCAAC*ttttttctcctttccc*
r11	10[352]	11[352]	ttttttttAAGGGTGAGAAAGGCCGTAGGTAAAGATTCAA*ttttttctcctttccc*
r13	22[348]	13[345]	ttttttttAGGTCACGTTGGTTCTAGCTG*ttttttctcctttccc*
r15	20[348]	15[345]	tttttttttTTAAATGTGAGCGCTATCAGG*ttttttctcctttccc*
r17	16[352]	17[352]	ttttttttTCATTTTTTAACCAATTTTTTGTTAAATCAGC*ttttttctcctttccc*
r19	18[352]	19[352]	ttttttttAAATTGTAAACGTTAAGTATAAGCAAATATTT*ttttttctcctttccc*
r21	19[322]	21[348]	GAAGATTTATTTTGCATTAAAAGGAACGTAGCCAGCTTTCATCAACA*ttttttctcctttccc*
r23	15[322]	23[348]	TACAAAGGAGTAACGGATTGACCGTAATGGGAT*ttttttctcctttccc*
l0	6[41]	0[15]	CCAACGCTCCCTTAAAGAGTCCACTATTAAAGA*ttttttctcctttccc*
l2	5[22]	2[15]	ttttttttCCGCCTGGCCCTCTGTTTGATGGTGGTTCCG*ttttttctcctttccc*
l4	19[22]	4[22]	ttttttttGCGAACTGATAGGATTGCCCTTCA*ttttttctcctttccc*
l6	3[15]	6[18]	ttttttttAAATCGGCAAAAGCGGGGAGA*ttttttctcctttccc*
l8	1[15]	8[18]	ttttttttACGTGGACTCCATTAATTGCG*ttttttctcctttccc*
l10	23[18]	10[22]	ttttttttAGCCAGGGTGGATGTTAAGCTTTACCGAGCTCACAATTCCAC*ttttttctcctttccc*
l12	9[18]	12[15]	ttttttttTTGCGCTCAGATAAAGACGGA*ttttttctcctttccc*
l14	7[18]	14[15]	ttttttttGGCGGTTTGGCATTTCACATA*ttttttctcctttccc*
l16	22[48]	16[22]	CAGTGCCCTTCTAATCCTTAGCCAAAATGGAGTGACTCTATGATACC*ttttttctcctttccc*
l18	17[42]	18[22]	CTGCCATGGCTATTAGTCTTTAATGC*ttttttctcctttccc*
l20	15[15]	20[18]	ttttttttAATCATTTCTCCTTGTCAACC*ttttttctcctttccc*
l22	13[15]	22[18]	ttttttttGGATCCCCGGGTCTCAGGAGA*ttttttctcctttccc*
A488	-	-	/5Atto488N/TGGGAAAGGAGAAAAAA

Bibliography

1. Carter, P.; Presta, L.; Gorman, C. M.; Ridgeway, J. B. B.; Henner, D.; Wong, W. L. T.; Rowland, A. M.; Kotts, C.; Carver, M. E.; Shepard, H. M. Humanization of an Anti-p185^{HER2} Antibody for Human Cancer Therapy. *Proc. Natl. Acad. Sci. USA* **1992**, *89*, 4285–4289.
2. Pan, Y.; Sackmann, E. K.; Wypisniak, K.; Hornsby, M.; Datwani, S. S.; Herr, A. E. Determination of Equilibrium Dissociation Constants for Recombinant Antibodies by High-Throughput Affinity Electrophoresis. *Sci. Rep.* **2016**, *6*, 39774.
3. Raghavan, M.; Bonagura, V. R.; Morrison, S. L.; Bjorkman, P. J. Analysis of the pH Dependence of the Neonatal Fc Receptor/Immunoglobulin G Interaction Using Antibody and Receptor Variants. *Biochemistry* **1995**, *34*, 14649–14657.
4. Ijäs, H.; Shen, B.; Heuer-Jungemann, A.; Keller, A.; Kostiainen, M. A.; Liedl, T.; Ihalainen, J. A.; Linko, V. Unraveling the interaction between doxorubicin and DNA origami nanostructures for customizable chemotherapeutic drug release. *Nucleic Acids Res.* **2021**, *49*, 3048–3062.
